# Supplementary material for: The Effect of Psyllium Husk on Intestinal Microbiota in Constipated Patients and Healthy Controls
Source: Int J Mol Sci. 2019 Jan 20;20(2):433. doi: 10.3390/ijms20020433 (PMC6358997; doi:10.3390/ijms20020433)
Supplement: Supplementary file 1 [file ijms-20-00433-s001.pdf]

Supplementary Material

The Effect of Psyllium Husk on Intestinal Microbiota in Constipated Patients and Healthy Controls

Jonna Jalanka [1,2], Giles Major [2], Kathryn Murray [2], Gulzar Singh [2], Adam Nowak [3], Caroline Kurtz [4], Inmaculada Silos-Santiago [5], Jeffrey M. Johnston [6], Willem M. de Vos [7] and Robin Spiller [2,*]

Table S1. Differences in the amount of short-chain fatty acids (SCFAs) during the intervention in both healthy and constipated. Significant difference against the baseline level (t-test, $p < .05$) indicated with an asterisk, standard deviation in brackets.

		Acetate (µM/g wet faeces)	Propanoate (µM/g wet faeces)	Butyrate (µM/g wet faeces)
Healthy controls	Baseline	6.90 (3.72)	5.49 (2.71)	5.88 (3.75)
	Psyllium 10.5g	8.68 (2.80)	7.16 (3.68)	6.17 (2.83)
	Psyllium 21g	8.86 (2.08)	7.12 (3.98)	5.58 (1.49)
	Placebo	11.56 (6.23)	8.42 (8.10)	8.36 (5.12)
Constipated	Baseline	21.65 (3.64)	4.70 (3.44)	5.32 (4.78)
	Psyllium 21g	**24.03* (2.75)**	**7.26* (3.47)**	6.87 (3.85)
	Maltodextrin	21.11 (3.44)	5.25 (4.15)	6.03 (4.99)
	Wash-out	21.74 (4.53)	5.09 (4.17)	5.54 (3.72)

Table S2. Associations between the intestinal microbiota composition and whole gut transit time (Weighted Average Position Score after 24 h (WAPS-24) and 48 h (WAPS-48)), relative stool water content and SCFAs, introduced by psyllium consumption in constipated patients. Each cell contains information of the direction of the association (+/-) and the FDR corrected p-value for this association. If the cell is empty, no significant association was found.

taxon	WAPS-48	WAPS-24	Water content	Propionate	Butyrate	Acetate
Actinobacteria; Actinobacteria; Actinomycetales; Actinomycetaceae; Actinomyces		+ , q=0.08	- , q=0.01			
Actinobacteria; Actinobacteria; Bifidobacteriales; Bifidobacteriaceae; Bifidobacterium			- , q=0.03			
Actinobacteria; Coriobacteriia; Coriobacteriales; Coriobacteriaceae; Asaccharobacter			- , q=0.02			
Actinobacteria; Coriobacteriia; Coriobacteriales; Coriobacteriaceae; Eggerthella		+ , q=0.005				- , q=0.05
Actinobacteria; Coriobacteriia; Coriobacteriales; Coriobacteriaceae; Slackia		- , q=0.05				
Actinobacteria; Coriobacteriia; Coriobacteriales; Coriobacteriaceae; uncultured		+ , q=0.09	- , q=0.01			
Bacteroidetes; Bacteroidia; Bacteroidales; Porphyromonadaceae; Odoribacter	- , q=0.02	- , q=0.03				
Firmicutes; Clostridia; Clostridiales; Christensenellaceae; Christensenella		+ , q=0.05	- , q=0.01	- , q=0.07		- , q=0.09
Firmicutes; Clostridia; Clostridiales; Clostridiaceae; Clostridium				- , q=5.69E-5		- , q=0.03
Firmicutes; Clostridia; Clostridiales; Eubacteriaceae; Anaerofustis		+ , q=0.08				
Firmicutes; Clostridia; Clostridiales; FamilyXIIIIncertaeSedis; IncertaeSedis				- , q=0.07		- , q=0.04
Firmicutes; Clostridia; Clostridiales; FamilyXIIncertaeSedis; Anaerococcus				- , q=0.02		
Firmicutes; Clostridia; Clostridiales; FamilyXIIncertaeSedis; Finegoldia				- , q=0.02		
Firmicutes; Clostridia; Clostridiales; Lachnospiraceae; Anaerostipes	+ , q=0.02					
Firmicutes; Clostridia; Clostridiales; Lachnospiraceae; Lachnospira			+ , q=0.03			+ , q=0.002
Firmicutes; Clostridia; Clostridiales; Lachnospiraceae; Roseburia			+ , q=0.03	+ , q=0.07		
Firmicutes; Clostridia; Clostridiales; Peptostreptococcaceae; IncertaeSedis	+ , q=0.07					
Firmicutes; Clostridia; Clostridiales; Ruminococcaceae; Faecalibacterium			+ , q=0.0001	+ , q=0.07		+ , q=0.04
Firmicutes; Negativicutes; Selenomonadales; Acidaminococcaceae; Phascolarctobacterium				+ , q=0.005		+ , q=0.005
Firmicutes; Negativicutes; Selenomonadales; Veillonellaceae; Veillonella						+ , q=0.02
Proteobacteria; Betaproteobacteria; Burkholderiales; Alcaligenaceae; Parasutterella			+ , q=0.03			
Proteobacteria; Betaproteobacteria; Burkholderiales; Alcaligenaceae; Sutterella	- , q=0.02	- , q=0.08				

Table S3. Associations between the intestinal microbiota composition and whole gut transit time (WAPS-24), relative stool water content and SCFAs, introduced by psyllium consumption in healthy subjects. Each cell contains information of the direction of the association (+/-) and the FDR corrected p-value for this association. If the cell is empty, no significant association was found.

taxon	WAPS-24	Propionate	Butyrate	Acetate	Water content
Bacteroidetes; Bacteroidia; Bacteroidales; Porphyromonadaceae; Parabacteroides			- , q=0.04		
Firmicutes; Bacilli; Lactobacillales; Leuconostocaceae; Leuconostoc			+ , q=0.03		
Firmicutes; Clostridia; Clostridiales; FamilyXIIIIncertaeSedis; IncertaeSedis				- , q=0.07	
Firmicutes; Clostridia; Clostridiales; FamilyXIIncertaeSedis; Anaerococcus					- , q=0.05
Firmicutes; Clostridia; Clostridiales; FamilyXIIncertaeSedis; Finegoldia			- , q=0.04		
Firmicutes; Clostridia; Clostridiales; FamilyXIIncertaeSedis; Peptoniphilus			- , q=0.09		
Firmicutes; Clostridia; Clostridiales; Lachnospiraceae; Blautia				+ , q=0.07	
Firmicutes; Clostridia; Clostridiales; Lachnospiraceae; Butyrivibrio		- , q=0.08			
Firmicutes; Clostridia; Clostridiales; Ruminococcaceae; Anaerotruncus				- , q=0.07	
Firmicutes; Clostridia; Clostridiales; Ruminococcaceae; Flavonifractor			- , q=0.0004	- , q=0.02	
Proteobacteria; Betaproteobacteria; Burkholderiales; Alcaligenaceae; Sutterella	- , q=0.05				
